# Case Report: Not a Mediastinal Mass! a Ruptured Giant Coronary Aneurysm That Occurred in a Young Man

**DOI:** 10.3389/fsurg.2022.812850

**Published:** 2022-03-18

**Authors:** Peng Yao, Cheng Shen, Zhi-Jie Xu, Yi-dan Lin

**Affiliations:** Department of Thoracic Surgery, West-China Hospital, Sichuan University, Chengdu, China

**Keywords:** rupture, mediastinal mass, giant, coronary artery aneurysm, young, case report

## Abstract

**Introduction:**

Coronary artery aneurysm (CAA) is a localized coronary artery dilatation that exceeds 1. 5 times the diameter of a standard adjacent segment or the largest coronary vessel. When the expansion is > 2 cm, it is called a “giant” coronary artery aneurysm. Giant coronary artery aneurysm rupture is extremely rare and fatal.

**Case presentation:**

We present a rare case of a 27 years old male with a giant coronary artery aneurysm rupture, but no catastrophic events occurred immediately. He was initially misdiagnosed as having a mediastinal mass with CT (computed tomography). The cardiac ultrasound showed no pericardial effusion. But The cardiac CTA (computed tomography angiography) showed a giant coronary aneurysm rupture with hematoma formation. He eventually underwent surgery and was followed up for 2 months without complications.

**Conclusion:**

We report this case of a ruptured giant coronary aneurysm because of its infrequent occurrence in coronary artery disease. It is tough to distinguish this disease from a mediastinal tumor, and chest MRI and cardiac CTA are crucial tests. Finally, surgical resection may be the right choice for coronary aneurysm rupture. More cases need to be reported to facilitate the preoperative diagnosis of this rare coronary aneurysm.

## Introduction

Coronary artery aneurysm (CAA) is a localized coronary artery dilatation that exceeds 1.5 times the diameter of a standard adjacent segment or the largest coronary vessel (1). When the expansion is > 2 cm, it is called a “giant” coronary artery aneurysm. Coronary aneurysms in patients undergoing coronary angiography range from 0.3 to 4.9% and are most often seen in the right coronary artery. The reason for CAA includes atherosclerosis, Kawasaki disease, congenital, arteritis, mycosis, connective tissue disease, entrapment, trauma (2, 3). Rupture of CAA is extremely rare, especially in a giant coronary aneurysm. Here we report a case of a young male patient who was diagnosed with a chronic rupture of a giant coronary aneurysm with hematoma formation in the left coronary artery and underwent surgical treatment.

## Case Presentation

**Figure 1:**
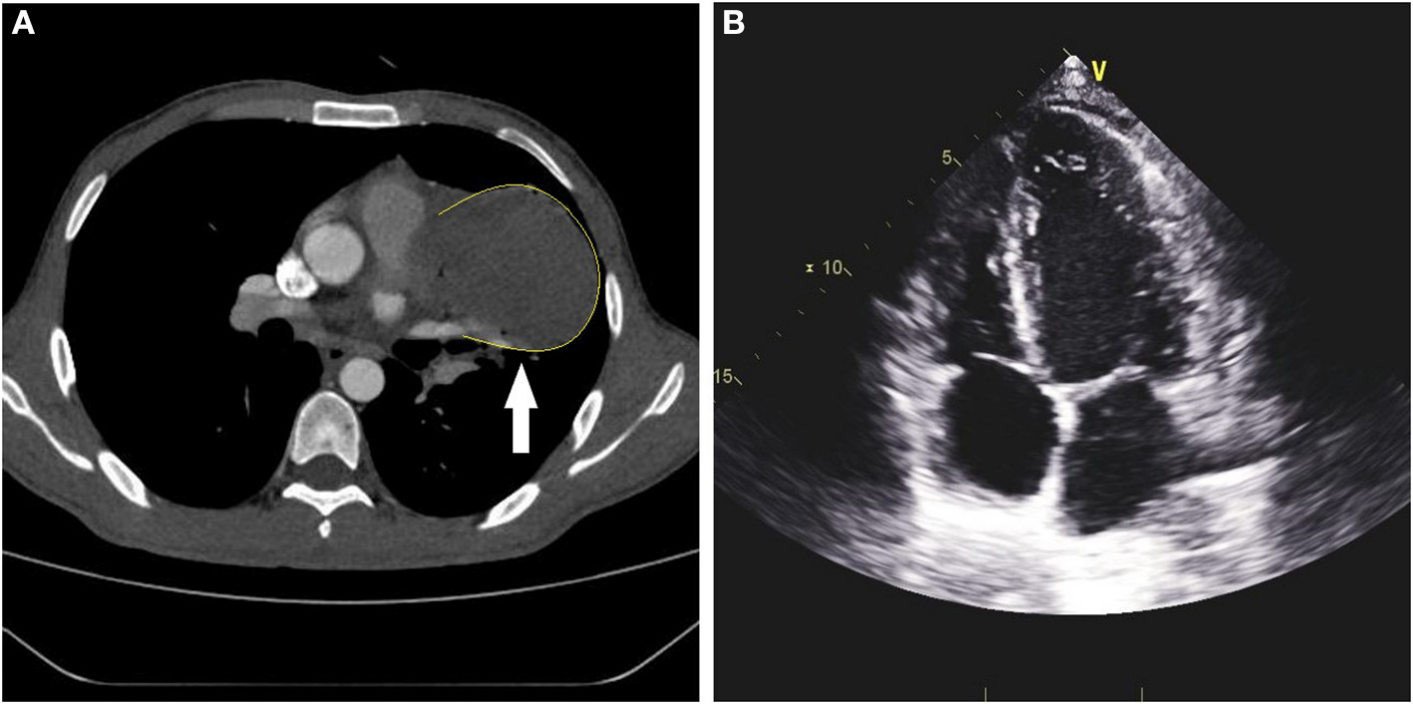
Preoperative chest CT and cardiac ultrasound. **(A)** Enhanced CT showed a 7.6-cm × 6.0-cm mixed-density mass shadow in the left parasternal pulmonary artery trunk of the middle mediastinum. **(B)** Cardiac ultrasound did not show pericardial effusion or structural abnormalities of the heart.

**Figure 2:**
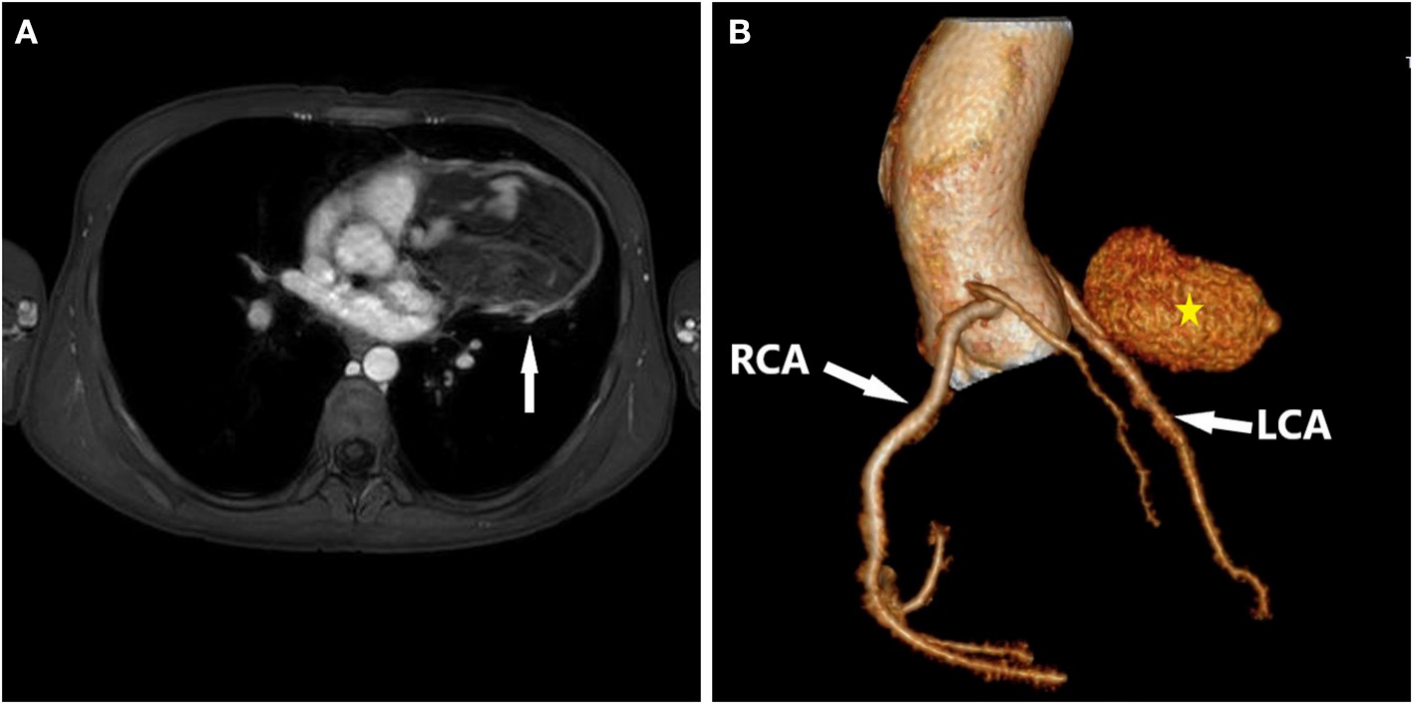
Preoperative chest MRI and Postoperative cardiac CTA. **(A)** MRI showed a large mixed-signal shadow in the left side of the middle mediastinum, measuring about 9.8 cm × 8.0 cm, with multiple patchy T1W1 high-signal shadows within it, and enhanced scans showed nodular enhancement in the lesion, measuring about 2.6 cm × 1.7 cm. **(B)** Preoperative cardiac CTA showed the middle segment of the anterior descending branch of the left coronary artery was poorly displayed, and a localized sac-like enhancement shadow was seen, with a maximum cross-section of about 2.6 cm × 1.7 cm.

**Figure 3:**
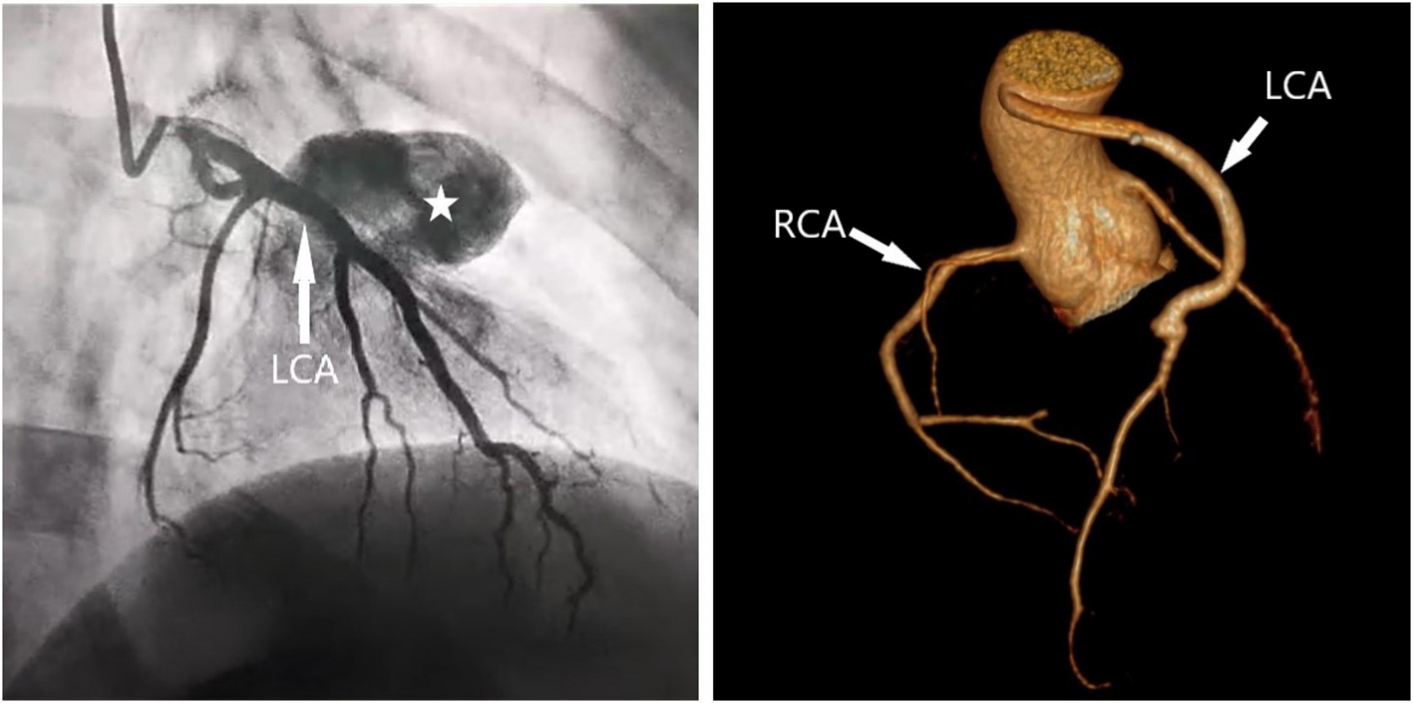
Preoperative coronary angiography and Postoperative cardiac CTA. **(A)** Preoperative coronary angiography showed a giant coronary aneurysm in the left anterior descending artery. **(B)** Post-operative CTA showed a favorable configuration of the left coronary artery.

A young 27-year-old male presented to our hospital with chest pain and hemoptysis. Previously, the patient had visited the local hospital several times for chest discomfort. Cardiac ultrasound and chest computed tomography (CT) did not show any abnormality, and the patient's symptoms did not resolve after conservative treatment. The cardiac ultrasound showed no pericardial effusion. At the time of this visit, the patient presented with intermittent chest discomfort for 4 months and hemoptysis for 3 days, and no prominent finding on physical examination. Subsequently, at our hospital, the chest CT showed a mixed density shadow of approximately 7.6 × 6.0 cm in size next to the left pulmonary artery in the middle mediastinum (Figure 1A); however, a subsequent cardiac ultrasound showed no pericardial effusion (Figure 1B), and previously he had no vascular risk factors or signs of systemic or inflammatory disease. ECG tests showed no abnormalities, and laboratory tests suggested the presence of mild anemia. Therefore, the nature of the left mediastinal mass is still unknown, and the tumor was not excluded. Considering the presence of hemoptysis, we performed fiber bronchoscopy to exclude lung tumors, and it still suggested negative findings. After careful evaluating the patient's history, a mediastinal tumor bleeding with hematoma formation was considered. Therefore, we further improved the chest Magnetic Resonance Imaging (MRI), which showed a large mixed-signal shadow in the left side of the middle mediastinum, measuring about 9.8 cm × 8.0 cm, with multiple patchy T1W1 high-signal shadows within it, and enhanced scans showed nodular enhancement in the lesion, measuring about 2.6 cm × 1.7 cm (Figure 2A). This result led us to the possibility of an aneurysm. Immediately after that, we performed a cardiac computed tomography angiography (CTA). It showed that the middle segment of the anterior descending branch of the left coronary artery was poorly displayed, and a localized sac-like enhancement shadow was seen, with a maximum cross-section of about 2.6 cm × 1.7 cm (Figure 2B). A subsequent coronary angiogram further confirmed the diagnosis (Figure 3A). The patient was eventually transferred to cardiac surgery for surgical treatment. The patient underwent surgery through a median sternotomy under cardiopulmonary bypass. The aneurysm was resected, and the saphenous vein was used as a bridging vessel to replace the resected portion of the left anterior descending branch. Intraoperatively, it was confirmed that the coronary aneurysm ruptured and compressed the left mediastinal pleura to form a massive hematoma, and the aneurysm formed adhesions with the mediastinal pleura, but no effusion was seen in the pericardium. Post-operative pathology of the coronary aneurysm wall showed vascular fibrosis and multifocal chronic inflammatory reaction. The patient was successfully discharged after seven days of hospitalization with no complications. Post-operative CTA showed a favorable configuration of the left coronary artery (Figure 3B). The patient was followed up for 2 months, and cardiac ultrasound and chest CT results were negative.

## Discussion

Patients with coronary aneurysms are usually asymptomatic. The reason for CAA includes atherosclerosis, Kawasaki disease, congenital, arteritis, mycosis, connective tissue disease, entrapment, trauma. Potentially severe complications, including rupture, thrombosis, embolism, entrapment, mechanical obstruction, and erosion of surrounding structures, are especially related to giant aneurysms (4). Rupture of coronary aneurysms is often etiologically related, and most of the ruptures are due to coronary atherosclerosis (5). In our patient, according to CT scan and histopathology of the resected tissue, we believe that this case was caused by vascular inflammatory factor and that Kawasaki disease could not be excluded (6).

Coronary artery aneurysm rupture is extremely rare and fatal. Acute coronary aneurysm rupture resulting in cardiac embolism, compression, coma, or even death has been reported in the literature (7–11). However, few reports of coronary aneurysm rupture did not result in a fatal event (12). This is the first report of chronic rupture of an aneurysm in young adults, which warned us that age should not be a factor in inferring the presence of coronary aneurysms, even in adults.

Young patients without any prior medical history often do not routinely undergo coronary angiography, making us vulnerable to blindness in diagnosing coronary aneurysms. In addition, the formation of hematoma after coronary aneurysm chronic rupture is extremely rare, and the preoperative cardiac ultrasound was negative, so the initial diagnosis of this case is highly likely to be misdiagnosed as a mediastinal mass or even lung cancer invading the mediastinum. Therefore, chest MRI and cardiac CTA are essential for occupying lesions of unknown nature in or adjacent to the mediastinum. The rarity of coronary aneurysms makes it difficult to standardize treatment or firmly establish guidelines to support optimal management. In the presence of severe complications, as in this case, surgical resection may be the right choice.

## Conclusion

There was no immediate catastrophic event in this ruptured case, but rather symptoms of compression caused by a pseudo-aneurysm. First, it is tough to distinguish this disease from a mediastinal tumor, and a negative cardiac ultrasound does not entirely exclude the possibility of coronary aneurysm rupture. Secondly, chest MRI and cardiac CTA are crucial tests for a young patient with a mediastinal tumor. Finally, surgical resection may be the right choice for coronary aneurysm rupture. More cases need to be reported to facilitate the preoperative diagnosis of this rare coronary aneurysm.

## Data Availability Statement

The original contributions presented in the study are included in the article/Supplementary Material, further inquiries can be directed to the corresponding author.

## Author Contributions

PY and CS was involved in drafting the manuscript. Y-dL designed and revised the manuscript. All authors have read and approved the final manuscript.

## Conflict of Interest

The authors declare that the research was conducted in the absence of any commercial or financial relationships that could be construed as a potential conflict of interest.

## Publisher's Note

All claims expressed in this article are solely those of the authors and do not necessarily represent those of their affiliated organizations, or those of the publisher, the editors and the reviewers. Any product that may be evaluated in this article, or claim that may be made by its manufacturer, is not guaranteed or endorsed by the publisher.
